# Age Differences in Attention Lapses Mask Age Differences in Memory Failures: A Methodological Note on Suppression

**DOI:** 10.3389/fpsyg.2013.00099

**Published:** 2013-03-01

**Authors:** James Allan Cheyne, Jonathan S. A. Carriere, Daniel Smilek

**Affiliations:** ^1^University of WaterlooWaterloo, ON, Canada

**Keywords:** age, attention failures, memory failures, statistical suppression

## Abstract

Although objective measures of memory performance typically indicate memory declines with age, self-reported memory failures often show no relation to age. In contrast, self-reported attention failures are reliably negatively correlated with age. This contrast suggests the possibility that age-related awareness and reporting of memory failures might be masked by a concurrent decrease in attention failures, which would reduce encoding failures with age and hence reduce perceived memory failures. Self-reported problems of attention and memory were evaluated in two samples with the ages spanning eight decades. Initial analysis indicated that attention failures significantly decreased with age, whereas memory problems did not to differ across age. The association of self-reported memory failures became significantly positive, however, when residualized on attention lapses. In contrast, the correlation between attention lapses and age was modestly affected when memory failures were controlled. These results highlight the close relation of attention lapses and memory problems and, beyond the implications of individual differences in attention for memory research, suggest the advisability of assessing attention failures for a full evaluation of memory problems.

## Introduction

One of the most robust findings in cognitive psychology concerns the decline with age in cognitive abilities, including speed of processing, selective attention, working memory, long term memory, and problem solving (Craik and Salthouse, 2008). Recent evidence suggests that one source of speed of processing slowing is an age-related increase in distractibility (Lustig et al., 2006). Older adults may have greater difficulty updating goals or suppressing no longer relevant information (e.g., Connelly et al., 1991; Hasher et al., 2007). All of these effects are consistent with findings of frontal lobe atrophy with age with increasing age (e.g., Raz, 2005). It is important to note that most studies reporting these age effects typically compare seniors with young adults and hence we are often ignorant of potential differences between either of these extreme groups and those of intermediate ages.

One apparent exception to the pattern of cognitive decline is absent-mindedness or mind-wandering. Against stereotype, older people report less mind-wandering and daydreaming in everyday life than younger individuals (Giambra, 1977–1978, 1979–1980; Singer and McCraven, 1961) and report less off-task thought during experimental tasks, including reading (Giambra, 1989; Smallwood et al., 2004; Jackson and Balota, 2012; Krawietz et al., 2012)^1^. That these reports validly reflect differences in cognitive functioning (see Giambra, 1989) is suggested by the superior performance of older compared to younger individuals on a behavioral test of failures of sustained attention, which were also related to self-reported absent-mindedness in everyday life (Cheyne et al., 2006; Carriere et al., 2010). It is important to note that this trend was observed across the entire adult life-span from late teens through the eighth decade (Carriere et al., 2010). It should be further noted that the improving sustained attention performance across age levels was largely accounted for by response speed. With increasing age through the adult life-span response speed slowed as performance improved. There was also evidence suggesting that the slowing of response tempo was beginning to have diminishing returns for improved performance among the oldest participants. It is therefore possible that the decrease in attention lapses with age is a secondary effect of a more measured or modulated response tempo with increasing maturity (see also Meyerson et al., 2007). Nonetheless, reduced reporting of attention problems with increasing age is intriguing for several reasons. Self-reported attention lapses and memory problems in everyday life are rather robustly positively correlated (Cheyne et al., 2006; Carriere et al., 2008) and declining memory ability is one of the most commonly reported cognitive effects of aging (Craik and Salthouse, 2008; Dixon et al., 2008). Memory declines are not, however, consistently found in self-report data (Erber et al., 1990; Cavanaugh, 1996). Moreover, self-reported memory abilities and failures have typically been found to be only moderately correlated with observed memory performance (Broadbent et al., 1982; Herrmann, 1982, 1984; Zelinsky, 1990; Hertzog et al., 2000; but see Herrmann et al., 2005; Rast et al., 2008). How is it that self-reported attention lapses are associated with relevant behavioral indices and show reliable age differences, whereas self-reported memory failures frequently fail to do either while still being positively correlated with attention failures?

Separating everyday attention and memory failures using self-report is challenging, as many cognitive failures in natural settings often reflect failures in multiple systems (Broadbent et al., 1982; Efklides and Sideridis, 2009). In particular, memory encoding is an attention demanding process (Craik et al., 1996; Naveh-Benjamin, 2001) and hence memory failure scan reflect encoding deficiencies during exposure to to-be-remembered events. Consistent with this line of reasoning, we have found self-reported failures of attention and of memory to be significantly moderately positively correlated and that the attention measures fully accounted for the simple bivariate association between self-reported memory failures and the behavioral attention tasks (Cheyne et al., 2006; Carriere et al., 2008). Jackson and Balota (2012) have also recently reported that older subjects made numerically fewer attention errors across three studies, though only significantly so in the third study. The weaker effects of errors compared to Carriere et al. (2010) likely reflect that the old group were somewhat older than the oldest in Carriere et al. study. Moreover, rather than comparing young adults to seniors, Carriere et al. employed a life-span sample and found that, among the older participants, increased slowing was producing diminishing returns in terms of improved performance. Such a finding is consistent with the finding that the older sample of Jackson and Balota showed a marginal advantage over young adults despite significant differences in response times.

The foregoing pattern of relations among age, attention lapses, and memory suggests the possibility of a type of statistical suppression (Horst, 1941). Horst’s (1941) treatment of suppression was rather specific. Classical statistical suppression occurs when a variable that is uncorrelated with a criterion, when added to a regression equation, increases the beta weight of another variable (Conger, 1974; Cohen and Cohen, 1975). Suppression was broadened somewhat by Darlington (1968) to include cases in which the final coefficient of one of the variables in a multiple regression equation is opposite in sign to the original. The term suppression has been broadened over the years to cover some of the complex ways in which predictors influence one another. Ultimately the term can be applied to any case in which the addition of a new predictor in a multiple regression analysis changes the weight of a predictor already in the equation in a surprising way. That is, the normal expectation of adding predictors is that, if they have any effect on other predictors at all, it will be to reduce and hence “explain,” the effect of a prior predictor. Surprising effects include an increase, in the same direction (i.e., enhancement), of the effect of a prior predictor, a change in the direction of the effect (from negative to positive or positive to negative), or an observed change such that a non-significant predictor becomes significant following the addition of the new predictor.

More formally, in predicting Y, the introduction of a third variable (X2) can change the regression beta, the partial, or the semi-partial correlation coefficient for the first predictor (X1) in several ways: it can (1) reduce the magnitude of a positive or negative coefficient, potentially to zero (variously referred to as partial or full mediation: Cheung and Lau, 2008; net suppression: Cohen and Cohen, 1975; confounding: MacKinnon et al., 2000; or redundancy: Paulhus et al., 2004); (2) increase the magnitude of a positive or negative correlation (classical suppression: Horst, 1941; Cooperative suppression: Paulhus et al., 2004); or (3) reverse the sign of a positive or negative correlation (analogous to Simpson’s paradox). In each case, there can be reciprocal effects on X2 (Conger, 1974). X1 and X2 can be single variables or separate linear combinations of different categories of variables (Tzelgov and Henik, 1985, 1991).

It is case 3 that is suggested by the attention-related encoding failure hypothesis. That is, if older adults experience fewer memory problems contingent on a reduction in mind-wandering episodes but more (inherent) memory problems otherwise, this combination would attenuate or even reverse the underlying positive correlation of age and inherent memory failures. This sort of suppression can occur when two strongly positively correlated variables have opposite bivariate associations with a third variable as in the case of attention, memory, and age reported above.

In the present study we compared self-reported attention and memory problems with aging in two independent samples. The first sample is from an archival data set including participants reported on previously in Cheyne et al. (2006, 2009) and Carriere et al. (2010), while the second consisted of newly collected data. Attention lapse self-reports were assessed by the Mindful Attention Awareness Scale – Lapses Only (MAAS-LO; Carriere et al., 2008). Memory failures were assessed by the Memory Failures Scale (MFS; Cheyne et al., 2006; Carriere et al., 2008). We expected to find negative correlations between age and attention measures and zero or weak positive correlations between age and reported memory failures. Importantly, we expected the age-memory association to be significantly positive once attention lapses are controlled. Such a result would provide evidence for a latent positive association between self-reported memory problems and age typically masked by a negative age-attention lapse correlations coupled with a robust positive correlation between attention lapses and memory failures. Such a finding would also contribute to an understanding of the previously observed unreliability of the relation of self-reported memory failures with age.

## Materials and Methods

### Participants

#### Sample 1

Participants were 766 individuals from an archival data set, including 516 females with a mean age of 40.67 (SD = 15.69; Range = 14–75) and 250 males with a mean age of 41.87 (SD = 16.65; Range = 15–85), who had previously participated in a web survey on sleep paralysis and indicated a willingness to participate in future research, and subsequently completed all three of the questionnaires. Each participant completed the study questionnaires on-line via the world-wide-web, and the order in which the questionnaires were presented was randomized across participants. The majority of these participants also completed the Sustained Attention to Response Task (SART; Robertson et al., 1997) after completing the questionnaires, the results of which have been reported on previously in Carriere et al. (2010).

#### Sample 2

Participants were 466 individuals from a new data set, including 276 females with a mean age of 41.41 (SD = 17.07; Range 18–89) and 189 males with a mean age of 41.13 (SD = 20.00; 18–82), who completed all the study questionnaires as part of a Human Intelligence Task (HIT) posted on the Amazon Mechanical Turk (www.mturk.com). This HIT also included additional pilot questionnaires on mind-wandering and fidgeting, which are not part of the present study, and the order of presentation of these questionnaires was randomized across participants. Following the questionnaires a subset of these participants also completed the SART. Participants were paid $1.50 for completing the HIT.

### Materials

The 12-item MAAS-LO (Carriere et al., 2008), a reduced form of the MAAS (Brown and Ryan, 2003), assesses attention lapses using a Likert scale ranging from *almost never* (1) to *almost always* (6), with higher responses indicating greater frequency of everyday attention lapses. Items refer to difficulty staying focused, finding oneself doing things without attending, carrying out activities (tasks and eating) automatically, feelings of “running on automatic.” The MAAS-LO has demonstrated good internal consistency, Cronbach’s α = 0.88 (Carriere et al., 2008). The MFS (Cheyne et al., 2006; Carriere et al., 2008) assesses the frequency with which one experiences everyday memory failures. Items cover such memory failures as forgetting birthdays and anniversaries people’s names, where one has placed things, what one intended to buy when shopping, as well as source memory and tip-of-the tongue experiences (for all items and their item-total correlations, see Carriere et al., 2008). The MFS is also a 12-item questionnaire employing a Likert scale of five possible responses, ranging from *never* (1) to *very often* (5) with higher scores indicating greater memory problems. The MFS has good internal consistency, Cronbach’s α = 0.85 (Carriere et al., 2008).

### Analyses

We assessed our predictions using semi-partial or part correlations rather than with regression or partial correlations as our basic interests were in determining the associations of each of the latent variables with age. Thus, memory scores were residualized on attention lapse scores and the memory residuals correlated with age to determine the association of memory failures with age rather than, as in the less conservative case of partial correlations or regression beta coefficients, only with that part of age variance not associated with the control variable.

## Results

Internal consistency, computed for all three scales for males and females separately for each sample: MAAS-LO, all Cronbach alphas > 0.87; MFS, all alphas > 0.82. These values are consistent with those observed in previous samples (Carriere et al., 2008). Pearson product moment correlation coefficients for age, attention, and memory are presented for males and females separately for each sample in Table 1. In three of the four analyzed samples, age was significantly negatively correlated with the MAAS-LO. The correlation of age and the MFS was, however, effectively zero in all four analyses. There were significant sex differences, which were small and inconsistent between samples. For sample 1, males produced slightly smaller means than females for the MAAS-LO, 3.13 (SD = 0.82) versus 3.31 (SD = 0.91), *F*(1, 764) = 5.73, *p* < 0.001, η^2^ = 0.02. For sample 2, males produced slightly larger means than females for the MFS, 2.67 (SD = 0.62) versus 2.54 (SD = 0.65), *F*(1, 461) = 5.17, *p* < 0.023, η^2^ = 0.01.

**Table 1:** **Pearson product moment correlations among age, memory (MFS) attention lapses (MAAS-LO), and sex**.

	Age	MAAS-LO	MFS
**SAMPLE 1**			
Age		**−0.26** (**−0.33**)	<0.01 (**0.20**)
MAAS-LO	**−0.20** (**−0.27**)		**0.63**
MFS	0.04 (**0.18**)	**0.55**	
**SAMPLE 2**			
Age		**−0.24** (**−0.27**)	0.03 (**0.15**)
MAAS-LO	**−**0.06 (**−0.14**)		**0.57**
MFS	0.09 (**0.16**)	**0.64**	

*Coefficients in parentheses are semi-partial correlations for residualized variables (see text). All coefficients in bold are significant (*p* < 0.05). Females above diagonal. Males below diagonal*.

Semi-partial correlations were calculated as follows: MFS scores were residualized on MAAS-LO and the residuals correlated with age. For both males and females in both samples, the coefficients changed from near zero values to significant positive correlations (Table 1; see coefficients in parentheses.) To assess deviations from linearity for each of these associations we examined second through fifth order age effects on MFS residual scores across both samples and none added significant prediction over the linear correlation. MAAS-LO scores were next residualized on MFS scores. The negative correlations of residualized MAAS-LO scores and age were slightly but not substantially improved in each of the analyses. In the one case in which MAAS-LO was initially non-significantly negatively correlated with age (males in Sample 2), the semi-partial correlation was now significantly negative. Thus the negative association of attention lapse scores with age was modestly affect by controlling for memory failures. We also assessed whether there were any higher order age effects beyond the linear correlation and none added significant prediction to the linear effect.

## Discussion

The results of the present study warrant several conclusions. First, we generally replicate previous findings reviewed in the Section “Introduction” that self-reported attention failures decrease with age. Second, before attention failures were taken into consideration, self-reported memory problems appeared not to differ systematically with age, also consistent with previous results. Third, and most critically, self-reported memory failures were found to be significantly positively related to age once attention lapses were taken into account. This effect was observed for both males and females in both samples and is consistent with the hypothesis of a degree of dependency of everyday memory problems on the ability to sustain attention during encoding. Individuals experiencing memory problems may do so because of intrinsic memory deficits of encoding, storage, and recall – or they may experience memory problems because of deficits involving attention lapses, which would mainly compromise encoding. Encoding failures lead to failures to remember events that people have reasonable expectations to remember.

### Some alternative interpretations

Given the present finding that, as individuals age/mature over the adult years, they report fewer lapses of attention in everyday life as well as behavioral evidence that they experience fewer attention lapses in laboratory tasks (Smallwood et al., 2004; Carriere et al., 2010; Jackson and Balota, 2012), we have argued that they will report fewer memory problems arising from attentional problems. This will have the effect of reducing their over-all score on the MFS, which may be matched, however, by a greater proportion of responses reflecting specific memory processes. Failure to detect increasing memory failures in everyday memory might also, however, reflect increased explicit strategic coping strategies, with increasing age involving more explicit and systematic attention to to-be-remembered materials. It is also possible, however, that the decrease in reported attention lapses may be externally scaffolded by others as one matures. It has been observed that others may, in interpersonal interactions, scaffold encoding for older adults with exaggerated prosody, simplified vocabulary, and less complex syntax, perhaps including subtle forms less obvious and patronizing and more useful than elderspeak (Kemper and Harden, 1999). Specifically, it is possible that as individuals mature they create and interact in interpersonal and organizational contexts that assist attention to task-related events and encoding of information. That is, the reduction in reported attention lapses with age need not solely reflect intrinsic changes in individuals but also to systematic changes in the living and working conditions that come with increasing age. There is, it should be acknowledged, likely also to be a more general bidirectional tendency to confuse and conflate attention and memory problems in self-reported cognitive problems as reflected in the somewhat larger coefficients for attention and age when controlling for memory complaints. There was some weak evidence to this effect in the small but consistent increases in the negative correlations between age and reported attention lapses.

### A caution: Adult life-span age difference versus aging

Rast et al. (2008) suggest the possibility that self-report may be affected by wide-spread stereotypes of the cognitive effects of aging. A common stereotype holds that older adults are forgetful and absentminded compared to younger adults (Heckhausen et al., 1989; Bolla et al., 1991; Hertzog et al., 1998; Derouesné et al., 1999). This stereotype is widely shared by young and old alike (Hertzog and Hultsch, 2000; Ponds et al., 2000; Zimprich et al., 2003) and applied to both self and others (McDonald-Miszczak et al., 1995; Cavanaugh et al., 1998). It is important to note that the present study is not simply a comparison of the very young and the very old (aging) but rather examines adult life-span changes over the adult years from the late teens to the 1970s. In the present case, the effects were observed across the adult life-span and not limited to the elderly, the functions being linear over the entire age range for the residualized memory and attention lapse variables, with no clear break in the post-retirement decades as would be predicted by the retirement-effect hypothesis, or also reasonably by a hypothesis that self-judgments might be based on stereotypes of old age.

## Conclusion

The present results clearly warrant cautious conclusions, being based on only two scales and two web samples. Nonetheless, given the empirical and theoretical interdependency of attention and memory in real world contexts, the present results should encourage assessments of memory, particularly surveys studies using self-report of memory, to be accompanied by evaluations of attention. Importantly, the present results also raise the possibility that compensatory strategies involving attention may not only mask but even effectively remediate memory problems to some degree. Beyond age differences, the present findings might also be taken to suggest that the study of any individual differences in self-reported memory failures consider controlling for individual differences in attentional problems that might affect memory performance, whether self-reported or objectively recorded.

## Conflict of Interest Statement

The authors declare that the research was conducted in the absence of any commercial or financial relationships that could be construed as a potential conflict of interest.

## Footnotes

^1^Intriguingly, the single study, of which we are aware, that failed to find an age difference in mind-wandering (Einstein and McDaniel, 1997) inferred mind wandering from a recall task.
